# Breaking Barriers: Exploring Neurotransmitters through In Vivo vs. In Vitro Rivalry

**DOI:** 10.3390/s24020647

**Published:** 2024-01-19

**Authors:** Gabriel Philippe Lachance, Dominic Gauvreau, Élodie Boisselier, Mounir Boukadoum, Amine Miled

**Affiliations:** 1 Department Electrical Engineering, Université Laval, Québec, QC G1V 0A6, Canada; gabriel.lachance.4@ulaval.ca (G.P.L.); dominic.gauvreau.1@ulaval.ca (D.G.); 2 Department Ophthalmology and Otolaryngology—Head and Neck Surgery, Université Laval, Québec, QC G1V 0A6, Canada; elodie.boisselier@fmed.ulaval.ca; 3 Department Computer Science, Université du Québec à Montréal, Montréal, QC H2L 2C4, Canada; boukadoum.mounir@uqam.ca

**Keywords:** lab-on-chip, organ-on-chip, microfluidics, microtechnologies, neurotransmitter, neurodegenerative diseases

## Abstract

Neurotransmitter analysis plays a pivotal role in diagnosing and managing neurodegenerative diseases, often characterized by disturbances in neurotransmitter systems. However, prevailing methods for quantifying neurotransmitters involve invasive procedures or require bulky imaging equipment, therefore restricting accessibility and posing potential risks to patients. The innovation of compact, in vivo instruments for neurotransmission analysis holds the potential to reshape disease management. This innovation can facilitate non-invasive and uninterrupted monitoring of neurotransmitter levels and their activity. Recent strides in microfabrication have led to the emergence of diminutive instruments that also find applicability in in vitro investigations. By harnessing the synergistic potential of microfluidics, micro-optics, and microelectronics, this nascent realm of research holds substantial promise. This review offers an overarching view of the current neurotransmitter sensing techniques, the advances towards in vitro microsensors tailored for monitoring neurotransmission, and the state-of-the-art fabrication techniques that can be used to fabricate those microsensors.

## 1. Introduction

Neurotransmitters have a crucial role in the functioning of the nervous system, which oversees the control and coordination of all bodily activities. Neurons synthesize neurotransmitters and store them in vesicles located at the ends of their axons. An action potential traveling down the axon reaches the axon terminal, prompting the release of the neurotransmitter into the synapse. Subsequently, the neurotransmitter diffuses across the synapse and binds to receptors on the postsynaptic neuron. This interaction can either enhance or inhibit the activity of the postsynaptic neuron.

Within the existing body of literature, numerous chemical compounds have been identified to function as neurotransmitters. Although numerous neurotransmitters have been identified, only a select subset is prominently involved in most well-known neuropathies, including Alzheimer’s, Parkinson’s, and Huntington’s diseases. These disorders are characterized by the deterioration of neuronal structure and function, which in turn influences specific neurotransmitter levels and the functioning of neurotransmitter systems.The precise identification and quantification of neurotransmitters are of great importance in neuroscience due to their crucial roles in the central nervous system and the severity of disorders associated with their dysfunctions.

Given the substantial differences in neurotransmitter compositions, standard detection techniques are often optimized for particular compounds, typically based on electrochemical or imaging approaches depending on the specific neurotransmitter under examination.

Numerous articles discussed the significance of neurotransmitter detection and described conventional approaches. These include well-established methods such as microdialysis and analytical chemistry-based techniques, as well as more recent research methods like optical and in vivo electrochemical sensing, which offer faster responses, higher sensitivity, selectivity, and instrument miniaturization. Classical techniques like microdialysis are established for clinical neurotransmitter sampling, while the use of newer techniques like optical sensing methods with nanomaterials is limited due to their less mature technology. The development of small, portable instruments for neurotransmission analysis has the potential to revolutionize the diagnosis and management of neurodegenerative diseases. These devices would be less invasive and enable continuous monitoring of neurotransmitter levels and activity in real time, providing earlier detection of disease progression and more targeted treatment interventions.

Recent advances in the field of microfabrication have led to the development of micro-instruments that could be used to study the dynamics of neurotransmitters in vivo. These instruments are based on microfluidics, micro-optics, and microelectronics and allow for the detection of neurotransmitters in a compact and easy-to-use manner. These micro-instruments offer several advantages over traditional methods of neurotransmitter detection. They are small, lightweight, and easy to use, making them suitable for use in clinical and laboratory settings. They also provide high temporal and spatial resolution, enabling real-time monitoring of neurotransmitter levels and activity with high sensitivity and specificity. Additionally, they are cost-effective and could potentially be used as a point-of-care diagnostic tool.

Micro-instruments for neurotransmission analysis based on micro-incubators for neurons could also be used for in vitro studies, providing an alternative to the more complex ex vivo and in vivo studies. In vitro studies are essential for understanding the fundamental mechanisms of neurotransmitter release, uptake, and metabolism. The micro-instruments can be used to measure neurotransmitter concentrations in microscale environments, allowing for the study of neurotransmitter dynamics in a controlled environment, which would also be more accessible than animal testing.

Overall, micro-instruments for neurotransmission analysis hold significant promise for the diagnosis and management of neurodegenerative diseases. They offer a non-invasive and continuous monitoring solution that could enable earlier detection of disease progression and more targeted treatment interventions. The development of these instruments is an exciting area of research, and further advances in microfabrication technology will undoubtedly lead to even more sophisticated and versatile devices for studying the dynamics of neurotransmitters in vivo and in vitro.

In this review, we introduce the current techniques used for the monitoring of neurotransmitters in vivo in Section 2. In Section 3, we introduce different advances that are being done in the fields of Lab-on-Chip (LoC) and Organ-on-Chip (OoC) that would change the way biochemistry research is being done. We also explore why these changes are required with the ever-changing politics and regulations in place that affect the world of research. We also highlight the techniques from Section 2 that would be suited to be integrated with those artificial organs to emulate the analysis that is being done in vivo. Finally, in Section 4, we introduce different novel microfabrication techniques and their usage for the fabrication of micro-incubators for in vivo neurotransmitter detection. This review is also intended to provide an overview of novel technologies that can be implemented in the field of neuroscience, technologies that might not have been correlated otherwise. We conclude this review in Section 5.

## 2. In Vivo Neurotransmitter Sensing Techniques

Within the current literature, a diverse array of methods exists for detecting neurotransmitters, each bearing distinct merits and limitations. These methodologies can be broadly categorized into five principal groups: nuclear, optical, electrochemical, sampling, and other techniques. Nuclear techniques involve the deployment of radioactive tracers, whereas optical techniques harness light to gauge neurotransmitter levels. Electrochemical techniques employ electrodes to quantify the electrical or chemical signals stemming from neurotransmitter release. Microdialysis entails the collection of extracellular fluid for subsequent analysis. Additional techniques encompass mass spectrometry, biosensors, and immunoassays. The selection of a specific technique hinges on the nature of the neurotransmitter under scrutiny and the intended purpose of the analysis. A comprehensive exploration of these distinct techniques has been undertaken by numerous authors in the past [1,2,3,4,5,6,7,8,9,10,11].

These reviews have primarily aimed to detail the range of techniques employed for detecting and analyzing specific neurotransmitters within the context of neurotransmission. Furthermore, they have engaged in comparative analyses, spotlighting the respective advantages and drawbacks of each technique.

In the scope of this review, we delve into the most recent advancements in conventionally reviewed neurotransmitter sensing techniques (between 2019 and 2023 inclusively). Additionally, we probe deeper into the realm of optics, exploring the potential for their miniaturization. This examination is particularly pertinent in light of a shifting research focus, wherein in vitro analysis takes precedence over in vivo analysis.

### 2.1. Medical Imaging Technique

Nuclear tomography neuroimaging techniques, namely positron emission tomography (PET) and Single-Photon Emission Computed Tomography (SPECT), offer insights into neurotransmitter release within the human brain. PET involves administering a radioactive substance that emits positrons into the patient’s bloodstream. The annihilation of a positron generates two gamma photons traveling in opposing directions, which a PET scanner detects, producing an image of radiotracer distribution. Conversely, SPECT entails introducing a radioactive agent emitting gamma radiation, selectively binding to specific tissues, thus highlighting distinct biological processes. Employing a gamma camera rotated around the patient, SPECT generates an image of the radiopharmaceutical distribution. Comprising a collimator to determine photon angles and gamma-ray detectors, the camera, along with a tomographic reconstruction algorithm, derives a three-dimensional map of radioactive activity.

Both PET and SPECT rely on competition between endogenous neurotransmitters and radiotracers to target molecules, scanning these radiotracers to construct images. PET boasts superior spatial resolution by employing two photons for pinpointing each point of interest. In contrast, SPECT emits a single gamma photon directly from the radioisotope atom, allowing heavier isotopes to label other molecules. However, PET surpasses SPECT in sensitivity, albeit at a higher cost, therefore enhancing spatial resolution.

Within the literature, PET has been utilized for imaging in the context of analyzing neurotransmission alterations in neurodegenerative disorders like Alzheimer’s disease [12,13]. Moreover, it has found applications in quantifying neurotransmitter dynamics [14] and exploring other pathologies [15].

In summary, both PET and SPECT are nuclear tomography neuroimaging techniques enabling neurotransmitter visualization within the human brain. Although PET offers superior spatial resolution, SPECT presents a cost-effective option that can make use of heavier isotopes for labeling. However, both techniques are constrained by developmental difficulties. Nonetheless, their sensitivity, selectivity, and spatial precision would render them a valuable goal in the pursuit of miniaturized LoC in vitro neurotransmitter sensing if there was a realistic way of reducing the size of such machines.

An approach adjacent to nuclear medicine that is amenable to conjoint use with PET is magnetic resonance spectroscopy (MRS). MRS operates on the foundation of magnetic resonance imaging (MRI), capturing radiofrequency signals emitted by diverse atoms in the brain. For neurotransmitter concentration assessment, researchers employ a specialized MRS technique known as proton magnetic resonance spectroscopy (^1^H-MRS). This technique exploits the distinct magnetic properties of protons, which vary based on their chemical environment.

MRS does not replace MRI techniques entirely; rather, it can complement other methods, such as PET. When paired with PET, for instance, MRS proves a potent approach for in vivo neurotransmitter analysis. In fact, the integration of MRI with PET (or analogous nuclear imaging techniques) has shown promise in gathering underlying neurotransmitter information [16,17].

On its own, MRS has demonstrated considerable efficacy in in vivo neurotransmission analysis [18,19,20,21,22]. However, owing to its foundation in MRI technologies, applying MRS to in vitro neurotransmitter detection is constrained by the technique’s complexity and the specialized instruments required.

### 2.2. Electrochemical Sensing Methods

#### 2.2.1. Voltammetry

Voltammetry stands as a robust electrochemical technique, wielding the capability to detect and quantify an array of analytes, including neurotransmitters, within biological specimens. Among its forms, fast-scan cyclic voltammetry (FSCV) emerges as a particularly efficacious means for probing neurotransmitter dynamics in the brain.

FSCV encompasses applying a modest voltage ramp to a carbon-fiber microelectrode immersed in a sample containing neurotransmitters. The progression of the voltage ramp precipitates shifts in electrochemical reactions at the electrode interface, resulting in a distinctive current that the system captures. Through the temporal analysis of current alterations, researchers can deduce the presence and concentration of specific neurotransmitters within the sample [3,23].

An important attribute of FSCV lies in its capacity to expeditiously cycle through numerous voltage ramps within a condensed timeframe, typically spanning milliseconds. This expeditiousness enables real-time tracking of neurotransmitter release and uptake, offering insightful glimpses into the operational mechanics of these molecules in the brain. FSCV’s repertoire extends to monitoring shifts in neurotransmitter release prompted by disparate stimuli, encompassing electrical and chemical cues, therefore augmenting its versatility.

Despite its many advantages, FSCV poses technical challenges, mandating specialized equipment and proficiency. Attentive calibration, meticulous control of electrode positioning, and conscientious management of other experimental variables are pivotal to procuring precise and reproducible outcomes. Nonetheless, with diligent training and a meticulous approach, FSCV evolves into a potent instrument for scrutinizing neurotransmitter dynamics in states both normative and pathological. Notably, the advent of advanced tools has demonstrated FSCV’s aptitude for detecting neurotransmitters at low concentrations [24,25,26,27,28]. Moreover, FSCV exhibits compatibility with other techniques, exemplified by its synergy with Raman Spectroscopy, culminating in a hybrid sensing platform that merges spectral and electrochemical data of the sample [29,30].

#### 2.2.2. Amperometry

Amperometry, an extensively employed electrochemical technique, holds prominence in neurotransmitter detection. It entails the application of a steady potential to a working electrode, measuring the resultant oxido-reduction reaction current, a measure proportional to the analyte concentration. The current flowing through the working electrode, in relation to a reference electrode, undergoes assessment. Of note, the temporal resolution of amperometry is not confined solely to the cycle duration, rendering it apt for scrutinizing neurotransmitter release kinetics.

To ascertain neurotransmitters, like catecholamines and indolamines, a carbon-fiber microelectrode assumes frequent use. This microelectrode maintains a voltage surpassing the oxidizing potential of the neurotransmitter. Upon neurotransmitter release, the emitted electrons are channeled to the electrode, producing a quantifiable current. The temporal resolution of amperometry contends with two factors: neurotransmitter diffusion towards the electrode and electron transfer kinetics.

The electrode-measured current hinges on the released neurotransmitter concentration, although the released neurotransmitter *per se* remains unidentified. To remedy this, a complementary approach, such as high-performance liquid chromatography, becomes requisite. Researchers bolster amperometry’s selectivity by modifying sensor surfaces with specific enzymes. Nevertheless, biological samples can introduce interference, sparking the evolution of numerous updated strategies.

Despite its merits, amperometry grapples with limitations: modest temporal-spatial resolution, susceptibility to interference in biological samples, and challenges in identifying precise neurotransmitters. Nonetheless, amperometry remains a popular method for neurotransmitter detection, as demonstrated by recently published papers [31,32,33,34,35,36,37].

#### 2.2.3. Electrochemical Sensing Methods for In Vivo or In Vitro Applications

Electrochemical sensing methods stand out as favored approaches for neurotransmitter detection. Their inherent simplicity and swift detection pace render them superior choices for neurotransmitter monitoring, especially in comparison to alternative techniques. Additionally, their adaptability proves noteworthy; by employing diverse electrode types and varying wavefronts, these methods can be tailored and refined to target specific molecules or environments effectively.

However, these techniques hinge on implantable electrodes that necessitate precise positioning and calibration to yield precise data. Ongoing endeavors by researchers are aimed at advancing implantable electrodes for in vivo measurements, exemplified by the work of Moldovan et al. [38], who presented a brain-implantable multifunctional probe for simultaneous detection of glutamate and GABA neurotransmitters. Nonetheless, we contend that the true potential of these techniques might lie in in vitro sensing, as demonstrated by their application preceding the shift towards in vivo objectives.

These methodologies could seamlessly integrate into miniaturized LoC in vitro neurotransmitter monitoring platforms, provided the electrodes are implanted into cells post-growth. Once inserted and accurately calibrated, these electrodes could persistently monitor neurotransmission processes, harnessing high temporal and spatial resolution in tandem with the technique’s selectivity and sensitivity.

### 2.3. Sampling and External Analytical Detection Methods

Microdialysis (MS) stands as a technique adept at sampling extracellular fluids, particularly within the brain, to gauge the concentration of diverse molecules, including neurotransmitters, peptides, and metabolites. Its implementation entails inserting a microdialysis probe into the desired tissue infusing it with a sterile physiological solution (the perfusate) at a gentle flow rate. This probe, featuring a semipermeable membrane at its tip, permits unhindered diffusion of small molecules between the perfusate and extracellular fluid.

As the perfusate courses through the probe, it equilibrates with the neighboring extracellular fluid, allowing molecules of interest to traverse the membrane into the perfusate. The collected perfusate subsequently undergoes analysis through various techniques to measure the target molecules’ concentrations. Remarkably, microdialysis lends itself to continuous extracellular fluid sampling in in vivo contexts, setting it apart from techniques restricted to singular or temporally limited observations [39]. Another advantage emerges from the ability to employ the same probe for introducing specific reagents at the measurement site, therefore inciting subsequent measurable reactions [40].

In the realm of neurotransmitter sensing, microdialysis facilitates the direct monitoring of neurotransmitter release at the synaptic level in response to diverse stimuli like drugs, stressors, or even neurodegenerative diseases. By tracking shifts in neurotransmitter concentrations over time, researchers glean insights into the regulation of neurotransmitter release and the underlying mechanisms governing neuronal activity. Moreover, microdialysis can assess neurotransmitter clearance from the extracellular space, furnishing information about neurotransmitter uptake and metabolism in the brain.

It is essential to note that since microdialysis merely extracts specific compounds from target tissues, external analyses are essential to process the obtained perfusate [41,42]. To bolster selectivity, researchers often integrate complementary techniques with microdialysis. Notably, high-performance liquid chromatography (HPLC) and capillary electrophoresis (CE) are frequently used to segregate neurotransmitters from microdialysis-obtained perfusate. Although both techniques serve neurotransmitter analysis, they diverge in mechanisms and analytical capabilities.

CE operates on the separation principle of charge and size differences, relying on a narrow capillary filled with a buffer solution through which an electric current passes. The sample is introduced at one capillary end, and components move along the capillary, separating based on electric field-induced movement. Highly sensitive and requiring minimal sample volumes, CE efficiently detects trace neurotransmitter quantities [11,43,44].

In contrast, HPLC hinges on separation according to chemical properties like hydrophobicity or polarity. Employing a column filled with a stationary phase, the technique segregates neurotransmitters and their metabolites in a biological sample, with high pressure propelling the sample through the column. Components are segregated based on their physicochemical attributes, resulting in a chromatograph with distinct peaks corresponding to different compounds. HPLC proves precise and adept at separating complex analyte mixtures, facilitating the identification and quantification of multiple neurotransmitters in a single sample.

These techniques excel in analyzing neurotransmitters due to their sensitivity and selectivity. When combined with microdialysis, they enable simultaneous measurement of multiple neurotransmitter concentrations, offering a comprehensive view of neurochemical shifts in the brain. Mass spectrometry (MS), another technique often amalgamated with microdialysis, deserves mention.

Mass spectrometry’s core principle involves ionizing a sample and detecting the resultant ions based on mass-to-charge ratios. For neurotransmitter analysis, molecules extracted via microdialysis are converted into gas-phase ions, followed by separation and detection according to mass-to-charge ratios using a mass analyzer. This technique provides a mass spectrum that charts ion abundance against mass-to-charge ratio, subsequently analyzed to identify and quantify target neurotransmitters. Software [45] programs can match sample mass spectra with a database of known neurotransmitter spectra, or selective detection of specific neurotransmitters can be achieved through multiple reaction monitoring (MRM), a targeted mass spectrometry technique.

In sum, mass spectrometry, characterized by high sensitivity and selectivity, proves invaluable for measuring neurotransmitters in biological samples. It has significantly contributed to neuroscience research, elucidating neurotransmitter roles in physiological and pathological conditions [46]. Microdialysis, coupled with external analytical methods, serves as a powerful tool for in vivo exploration of neurotransmitter release and clearance, extensively employed in neuroscience research to delve into the neurochemical basis of behaviors and diseases. Although these techniques are useful, they cannot be entirely miniaturized within LoC neurotransmitter sensor contexts, as other analysis techniques are still needed and cannot be fully miniaturized in their current forms. An alternative approach, as presented by Lendor et al. [47], entails using solid-state microextraction probes for local sampling and subsequent external analysis. The possibility of coupling microdialysis with solid-state microextraction probes within LoC technologies as a method of sampling neurotransmitters and utilizing separate analysis techniques amenable to miniaturization holds potential and is further described in the subsequent section.

### 2.4. Optical Sensing Methods

#### 2.4.1. Fluorescence

Fluorescence detection revolves around measuring the fluorescence emitted by specific molecules when subjected to particular light wavelengths. However, neurotransmitters are not inherently fluorescent, necessitating their labeling with a fluorescent group to enable detection. The fluorescence intensity directly corresponds to the concentration of the target neurotransmitter. Adjacent to direct fluorescence measurements, techniques such as Förster Resonance Energy Transfer (FRET) and Photoinduced Electron Transfer (PIET) are employed, relying on changes in the fluorescence signal. Detection occurs by gauging fluorescence quenching in the presence of an acceptor molecule.

Fluorescence measurements can be combined with various techniques to detect neurotransmitters. On its own, fluorescence provides information on neurotransmitter concentration at the measurement site. Yet, coupling fluorescence with imaging techniques imparts spatial information alongside concentration data. For instance, fluorescence microscopy permits imaging of a region of interest while accurately assessing neurotransmitter concentration within it. This approach is useful for studying neurotransmitter release and uptake in live cells or tissues. Notably, these labeling techniques are mainly suited for in vitro applications due to the potential biological incompatibility of the labeling chemicals and the ease of measuring fluorescence signals using advanced high-resolution microscopes. Fluorescence measurements have yielded success in a plethora of in vitro research studies [48,49,50,51,52,53,54], with some instances of in vivo success [55,56].

Fluorescence imaging boasts high specificity, as well as temporal and spatial resolution [57]. It has been proven effective for neurotransmitter detection in ex vivo tissues. Yet, for the imaging aspect, in vivo use is constrained due to compatibility issues with suitable imaging techniques. Employing a fluorescence probe could facilitate in vivo measurements at the cost of some spatial resolution, although advancements in compact and miniaturized imaging technologies might render fluorescence measurements feasible for in vitro neurotransmitter detection within LoC platforms.

#### 2.4.2. Chemiluminescence

Chemiluminescence is a detection technique involving the emission of light resulting from a chemical reaction. Much like fluorescence detection, this technique can employ either direct intensity measurement or differential measurement. When the chemical reaction directly involves the molecule of interest, the measured light intensity can be directly linked to the molecule’s concentration. Conversely, in cases where the molecule does not actively participate in the reaction, its presence can partially inhibit the reaction, causing a reduction in the resulting light production. The disparity in light intensity with and without the molecule’s presence correlates with its concentration.

Similar to fluorescence measurement, chemiluminescence measurements can achieve high sensitivity and selectivity, yet they necessitate supplementary equipment and should be paired with other techniques for optimal selectivity outcomes. This technique is commonly employed for in vitro analysis due to the chemical agents used and the detection methods not aligning well with in vivo applications. Recent strides in the field of chemiluminescence neurotransmitter sensing primarily showcase their results through in vitro analyses [58,59,60,61,62,63,64].

#### 2.4.3. Optical Fiber Biosensor

Optical fibers are extensively utilized in various biodetection applications. They can serve as imaging mediums when combined with other detection techniques, but in scenarios involving direct detection of neurotransmitters, optical fibers can be modified to function as standalone detection vectors, especially when coupled with advanced silicon light-emitting device (LED) as their light source [65].

Through tapering the end of an optical fiber to a fine point, the light propagating within its core interacts with the surrounding medium via the evanescent field. Coating the tapered end with a reactive compound transforms the optical fiber into a detection probe.

In one approach, the optical fiber’s tapered end is coated with fluorophores. The evanescent field generated by the fiber excites the coated fluorophores, with the resulting emission being propagated a short distance in the surrounding medium. This setup allows for precise spatial measurements of the target molecules [66,67].

Another method involves coating the optical fiber with aptamers. Here, detection involves measuring the light transmitted and reflected within the optical fiber. Specific aptamers bind with the target molecule, inducing a change in their conformation. This alteration in the aptamer’s structure affects the refractive index of the medium surrounding the optical fiber, modifying its transmission properties. Zhu et al. demonstrated a similar concept using gold and molybdenum disulfide nanoparticles functionalized with acetylcholinesterase instead of aptamers, enhancing sensitivity [68,69].

Given their thinness, optical fibers minimally disturb the medium in which they are employed, making them suitable for in vivo probing [70]. They can also be employed for ex vivo or in vitro measurements for the same reasons. Although the equipment and techniques associated with optical fiber biosensors are less intricate compared to others, enabling the miniaturization of biosensors, the trade-off lies in the spatial measurements, as individual optical fibers interact with limited volumes of the medium at a time.

#### 2.4.4. Electromagnetic Detection

Two key techniques arising from the electromagnetic theory of photon-matter interactions are employed to detect and identify molecules.

First, Surface-Enhanced Raman Spectroscopy (SERS) enhances Raman scattering through molecule-surface interactions. This phenomenon stems from the excitation of localized surface plasmon resonance in surface molecules upon exposure to electromagnetic emission. The resonance frequency alters when the target molecule interacts with the surface, yielding composition information. SERS can enhance Raman scattering by up to 10^10^ times, enabling single molecule detection. It offers high sensitivity and specificity and facilitates multiplexed detection of multiple neurotransmitters concurrently. For optimal sensitivity and selectivity, functionalized surfaces are crucial for each molecule. These surfaces often involve nanostructures like gold or silver nanorods/nanoparticles to generate high electromagnetic fields. To further enhance the technique’s effectiveness, plasmonic–magnetic silica nanotubes and aptamer-functionalized nanoparticles are used to augment the enhancement factor.

Although SERS promises impressive sensitivity and selectivity, it requires specialized and complex equipment. Nonetheless, it remains a widely popular technique for neurotransmitter detection [30,71,72,73,74,75,76,77,78,79,80,81,82,83,84,85,86,87,88,89,90,91,92,93,94,95,96,97,98,99,100,101]. In recent works, research teams are exploring implantable SERS-based nanoprobes for direct neurotransmitter detection in the brain [69,102,103,104,105].

On the other hand, Localized Surface Plasmonic Resonance (LSPR) leverages the plasmonic resonance of nanostructure colloids to detect molecules of interest. Like SERS, this technique relies on molecule-nanostructure interactions, leading to changes in plasmonic resonance. However, here, nanostructures are dispersed in a fluid colloid rather than being confined to a surface. Detection in LSPR does not hinge on Raman scattering; instead, it relies on colorimetry analysis. The base plasmonic resonance of nanostructure colloids results in specific resonance absorbance in response to incoming electromagnetic spectra. This absorption band, called the plasmonic resonance frequency, shifts upon the interaction of the molecule of interest with the nanostructures. This shift provides concentration information. To amplify this interaction, nanostructures can be functionalized with aptamers or antibodies, strengthening the interaction and increasing the plasmonic resonance band shift.

In contrast to SERS, LSPR detection is accomplished using simpler devices. Since colloid plasmonic resonance band analysis essentially involves colorimetry, standard spectrophotometry instruments suffice. Miniaturized optical instruments facilitate the implementation of LSPR in LoC setups and miniaturized implantable devices. However, both LSPR and SERS techniques share a limitation: they often rely on toxic substances in the form of metallic or nanostructure colloids, making them unsuitable for in vivo analysis. To address this, research focuses on developing biocompatible, functionalized ultrastable nanoparticles, aiming to design an LSPR technique that, in conjunction with miniaturized devices, can be employed in vitro or as implantable sensors [68,106,107,108,109,110,111,112,113].

### 2.5. Comparison of Sensing Methods

In the previous sections, many different neurotransmitter sensing techniques were outlined. Those techniques are all sensing methods used and found in today’s literature. Table 1 lists those methods and highlights their ease of use.

Finding an objective comparison for all cited methods is challenging. For example, a technique that is superior in sensitivity can also be inferior in ease of use or not as selective as another. The comparison and the identification of the superior techniques all depend on the specific intent of the user. In this review, we compare the efficiency of the techniques based mainly on ease of use as well as its miniaturization potential. Of course, the sensitivity and selectivity of the measurements are important. However, the usefulness of a sensing technique is not solely based on the sensitivity or selectivity as it depends on the use case. A better comparison basis would be the usability of the technique for a specific purpose. The definition of usability of a process or system, as defined by the International Organization for Standardization (ISO) [ISO 9241-11:2018] [117], is a combination of effectiveness (the accuracy and completeness with which specified users can achieve specified goals in particular environments), efficiency (the resources expended in relation to the accuracy and completeness of goals achieved) and satisfaction (the comfort and acceptability of the work system to its users and other people affected by its use) of the process or product in question. Consequently, the usability of each neurotransmitter sensing technique depends on the application. It is thus difficult to compare all the sensing techniques together to identify the superior one with an objective approach. In the scope of this review, we focus more on the miniaturization potential and the potential for its application to an in vitro instrument.

In that sense, we can compare techniques together based on their in vivo or in vitro usage.

#### Comparison of Sensing Methods

In vivo measurements provide valuable insights into the dynamics of neurotransmitter release and uptake in their natural physiological and pathological contexts. They allow researchers to observe how neurotransmitters function in living organisms, which is crucial for understanding the complex processes that occur in the body. However, in vivo measurements come with significant challenges, including the complexity of the environment, low neurotransmitter concentrations, potential interference, and variations between individual organisms.

In vitro measurements, on the other hand, offer a more controlled and reproducible environment. Researchers can manipulate variables and obtain precise measurements of specific neurotransmitters. This controlled environment can lead to higher sensitivity and specificity in the measurements. However, in vitro measurements may not fully capture the complexity and dynamics of in vivo activities, and the results may not directly apply to living organisms.

To address the need for more compact and autonomous instruments for neurotransmitter detection, there is potential to shift towards in vitro measurements. Microfabrication technologies can play a crucial role in miniaturizing existing detection techniques. Although some techniques, like PET scanners, may not be cost-effective to miniaturize for in vitro use, others, such as fluorescence, chemiluminescence, optical fibers, LSPR, amperometry, voltammetry, and microdialysis combined with HPLC or CE methods, show promise for miniaturization in the context of in vitro neurotransmitter detection instruments.

By miniaturizing these techniques and creating LoC or small-scale in vitro sensors, researchers can benefit from controlled environments, reproducible results, and the ability to manipulate and measure specific neurotransmitters with high precision. This approach can complement in vivo measurements and provide valuable insights into neurotransmitter function, particularly in drug development, neuroscientific research, and clinical diagnostics.

Ultimately, the choice between in vivo and in vitro measurements should align with the specific research objectives, and a combination of both approaches may offer the most comprehensive understanding of neurotransmitter dynamics and their role in various physiological and pathological conditions.

## 3. The Future of Neurotransmitter Sensors

As was presented previously, many different technologies already exist for the detection of neurotransmitters both in vivo and in vitro, which uses instruments ranging from extremely bulky and encumbering to compact, portable, and even miniature. We believe that the next generation of neurotransmitter sensing instruments will stand with the design of even more compact and miniature instruments as they are both easier to use generally and less costly than their larger counterpart. The potential benefits of such miniaturized instruments are significant, including increased accessibility, reduced costs, and more efficient data collection and analysis.

However, the development of high-sensitivity and high-selectivity miniature neurotransmitter sensing platforms does come with its challenges. Achieving the required level of sensitivity and selectivity while also ensuring reproducibility and reliability can be complex. Researchers need to consider factors such as sensor design, material selection, integration of different techniques, and optimization of signal-to-noise ratios.

By creating a bridge between in vivo and in vitro measurements, researchers could potentially overcome some of the challenges associated with in vivo measurements, such as variability between subjects and interference from the biological environment. These in vitro platforms could serve as valuable tools for validating the accuracy and reliability of in vivo measurements, providing complementary insights into neurotransmitter dynamics.

As with any technological advancement, collaboration between scientists, engineers, and researchers from various fields will be crucial in achieving success. Developing novel miniature in vitro platforms for neurotransmitter sensing requires interdisciplinary efforts, combining expertise in materials science, microfabrication, biology, and neuroscience.

Furthermore, an additional factor supporting the necessity for a new generation of small-scale sensors, such as LCOs or other in vitro instruments, lies in the ongoing transformations within the domain of live animal testing. Evidently, the recent prohibition of live animal testing within the cosmetic sector has triggered a shift in the practices of Canada’s regulatory authorities, aligning with a broader global trend steering away from live animal utilization in research endeavors [118,119,120,121]. This trajectory, initially embraced by pioneering nations including the United Kingdom [122], the European Union [123,124,125,126], and the United States [127,128,129], heralds a transformation that, in our estimation, will redefine the landscape of research methodologies in the future. Although presently confined to specific domains, we anticipate that these regulatory shifts will exert a profound influence on the broader research landscape. In this regard, the exploration of alternatives that circumvent the need for live animal or human testing becomes imperative.

Another pivotal dimension driving the transition from in vivo investigations to in vitro studies is the growing apprehension among scientists regarding the accuracy of animal models vis-à-vis human models. In essence, a cadre of researchers has underscored instances where animal models have proven inadequate in faithfully replicating observations subsequently witnessed in analogous human models within the same studies [130,131,132]. These discrepancies have manifested across diverse fields of study, spanning disciplines such as toxicology [133], pathology [134,135], and neurology [136], among others. In response to this predicament, a novel paradigm is being advocated, a paradigm that resembles closer to human models while veering away from animal models that have demonstrated suboptimal fidelity to their human counterparts.

Our stance pivots on the recognition that animal models may, in certain instances, lack fidelity with human models across diverse fields of study. However, animal models retain their pertinence in specific contexts and, provided they are employed judiciously and ethically, continue to constitute a valuable resource in the scientific pursuit of human advancement.

A novel research trend has emerged in response to the aforementioned regulations and concerns, aiming to discover alternatives to animal experimentation. This research avenue is dedicated to exploring New Approach Methodologies (NAMs) that can yield equivalent outcomes to those achieved through live animal experimentation. In pursuit of this objective, various scientific journals [137,138] and conferences [139] have been established, providing platforms for scientists to showcase their progress in NAMs and related in vitro measurement techniques. Researchers in this field are directing their efforts towards substituting traditional in vivo analysis methods with alternatives, therefore mitigating the reliance on animal subjects. They are devising innovative means to attain comparable outcomes by harnessing in vitro, in chemico, and computational (or in silico) strategies [140,141,142,143].

The adoption of LoC and OoC devices has gained prominence within these alternatives, aiming to replace live subjects in experimentation [144,145]. This evolution emphasizes the importance of adapting existing techniques for application within such devices to shape the future landscape of research. In this regard, a prudent approach involves the adaptation of established in vitro techniques that have proven useful while identifying viable candidates among in vivo techniques that can be transposed to an in vitro context. In the preceding sections, a comprehensive overview of various techniques was presented alongside recent applications. In the subsequent section, we will evaluate each technique to identify those that align most favorably with LoC and OoC devices while also identifying those less suited for in vitro measurements.

### 3.1. Envisages Miniaturized Hybrid Neurotransmitter Analysis Platform/Device

As the era of in vivo measurements gives way to a growing emphasis on in vitro approaches, a novel class of instruments is required to provide researchers with the capacity to advance our understanding of neurotransmitters. To do so, a new generation of neurotransmitter in vitro measurement devices becomes necessary.

In this context, we propose LoC and OoC-based instruments as the new generation of sensors. These cutting-edge devices are currently undergoing development, progressively augmenting their capabilities and poised to supplant animal and human models in the realm of biochemical analysis [146]. By offering an in vitro counterpart to animal or human models, LoC and OoC devices offer an alternative avenue to in vivo analysis [147]. This paradigm is especially advantageous for disciplines necessitating specific conditions or model characteristics that are challenging to replicate in in vivo animal or human models but are more controllable within artificial in vitro constructs. This holds for disease investigations and diagnostics, where the versatility and control offered by these devices prove invaluable [148,149,150,151,152]. Moreover, such platforms furnish a controlled and reproducible environment, ideally suited for the nuanced exploration of intricate processes and delicate organs, such as the brain [153,154,155].

For studies focused on neurotransmitters, the integration of LoC and OoC technologies emerges as highly favorable. These hybrid platforms (integrating LoCs with OoCs on the same platform) enable researchers to cultivate precise conditions and harness specific neurons vital to their analyses, all within a controlled and replicable environment [156,157,158,159,160]. The ability to embed or implant probes for neurotransmitter measurements is greatly facilitated, owing to the comprehensively regulated parameters that characterize these platforms, an achievement that remains difficult in traditional in vivo investigations.

To create a viable LoC or OoC platform capable of ultimately supplanting in vivo analysis, several essential requisites must be fulfilled. First, the device must faithfully emulate the subject of interest, mirroring the attributes of the animal or human model. In pursuit of this aim, the utilization of organelles or cultivated biological constructs has emerged as an apt approach for such investigations [146]. To nurture and sustain this biological matrix within conditions akin to in vivo environments and to manipulate the matrix without compromise, a microfluidic framework stands as a promising solution. The inherent attributes of microfluidics facilitate the controlled infusion and circulation of fluids containing the requisite components for cellular growth and viability in vitro. Furthermore, this technology enables the precise delivery of reagents and the extraction of samples for subsequent analyses, all while preserving the integrity of the biological matrix. The efficacy of microfluidic platforms for LoC and OoC devices tailored to in vitro neuronal analyses has already been demonstrated [161,162,163,164].

The integration of a judiciously chosen miniaturized sensing approach and system is pivotal for the intended analysis within such a device. For instance, in the context of neurotransmitter detection, different choices of miniaturized analysis methods can be highlighted from the existing methods highlighted in Table 1. The reasoning of such a choice will be explored in the following subsection.

A pivotal requirement for all LoC and OoC devices resides in their physical dimensions. To truly rival the prevailing in vivo paradigm, the LoC and OoC-based NAMs must present more than a mere alternative; they must provide a distinctive advantage. A prominent asset intrinsic to these designs is their potential for compactness and miniaturization, a characteristic that can be harnessed to offer unparalleled convenience. A technology that can be used to help with the compact nature of such devices is 3D printing. Remarkably, the application of 3D printing technologies extends beyond the fabrication of mechanical components, encompassing the creation of microfluidic components as well [165,166,167]. The advancements in printer resolution have enabled the direct printing of intricate microfluidic channels within systems, therefore allowing for more streamlined and integrated configurations [168,169]. Notably, recent investigations have further harnessed 3D printing technologies to engineer biological matrices, therefore crafting two-dimensional or three-dimensional cellular frameworks for integration within LoC or OoC devices [163,170,171].

### 3.2. Existing Neurotransmitters Sensing Methods That Could Be Used for In Vitro Miniaturized Sensing Methods

In Section 2, we explored the existing neurotransmitter sensing methods and how they were best used. However, in the context of the future shift from in vivo to in vitro testing, we should decide which existing technique is best suited for this paradigm shift. Table 1 provided a review of the different techniques and their usage.

The first methods under consideration were medical imaging techniques, including MRI, MRS, PET, and SPECT. These techniques often involve in vivo imaging and are adept at examining the internal structures of specimens with remarkable spatial accuracy. However, the complexity of the techniques and the instruments required to generate these images is significant, and they are typically designed to accommodate human subjects. Although some miniaturized versions have been developed to accommodate smaller specimens, such as live mice [114,115,116], they are not commonly utilized. For the application of medical imaging techniques in in vitro neurotransmitter analysis, the development of a miniaturized version, ideally designed to fit around a Petri dish or similar container, would be necessary. Nonetheless, the challenges associated with miniaturization would persist, rendering this approach one of the more formidable to integrate into a compact LoC or OoC device.

The second set of techniques highlighted are electrochemical sensing methods, encompassing voltammetry and amperometry. These methods offer a relatively simpler sensing approach. They are rooted in the examination of the current response of a chemical compound under an applied potential, whether stable or variable. This entails the use of an electric probe, a variable power source, and a current meter. The data analysis necessary for the detection of the target chemical, such as neurotransmitters in this context, is comparatively straightforward, particularly when juxtaposed with more intricate methods like medical imaging. Furthermore, the probes employed can be fabricated at the microscopic scale using contemporary techniques, utilizing materials like carbon nanotubes or nanodots [25,34,35,36]. These electrochemical techniques have demonstrated success both in vivo and in vitro, with in vitro experiments often serving as a stepping stone for in vivo applications. Their relative ease of implementation makes them particularly suitable for establishing proof-of-concept in in vitro contexts, paving the way for future in vivo investigations. As a result, these methods are well poised for adaptation into a miniaturized in vitro neurotransmitter sensing platform built upon LoC or OoC architectures.

The third approach was microdialysis, a distinctive technique that deviates from direct neurotransmitter detection. Functioning more as a sampling method than a standalone detection technique, microdialysis extracts neurotransmitters from the analyzed sample. Although microdialysis is categorized here as a detection technique due to its integration with methods like HPLC for neurotransmitter analysis, its primary function is the extraction of the target molecules from the sample. This technique is primarily employed for in vivo neurotransmitter sampling, facilitating the collection of specimens for subsequent external analysis. In the context of in vitro analysis, microdialysis does not inherently hold a primary role, as many in vitro analyses do not necessitate extensive sampling. However, its integration into OoC or LoC devices could be envisaged. OoC or LoC devices might require sampling for external analysis of the molecules of interest. The miniaturization of microdialysis has been accomplished, yielding compact dialysis probes capable of extracting sample volumes as minute as nL. When coupled with an OoC or LoC device, this miniaturized microdialysis could potentially enable robust in vitro neurotransmitter analysis. This approach could be particularly effective if combined with a complementary secondary analysis method, such as HPLC.

The fourth method encompasses chemiluminescence techniques, including both fluorescence and chemiluminescence-based detection. Fluorescence and chemiluminescence are primarily suited for in vitro analysis, given that the reactive compounds employed in these methods often possess toxicity that makes them ill-suited for in vivo applications. In some instances, fluorescent dyes have been utilized in vivo when bound to nanoparticles, serving as the detection vector for these nanoparticles. This combination has demonstrated certain degrees of biocompatibility, enabling in vivo analysis. However, for the ease of use and reduced concern over biocompatibility, employing the fluorescence dyes or chemiluminescent reagents directly in in vitro analysis is preferable. These chemiluminescence-based analysis methods align well with the notion of a miniaturized neurotransmitter detection platform integrated with LoC or OoC devices. Their integration would not compromise the compact dimensions of these devices. The primary requirement here would be to incorporate suitable sensing techniques to capture the luminescence or fluorescence produced by the compounds in the presence of neurotransmitters. Integrating these sensing techniques into the LoC or OoC device would then create a synergistic in vitro neurotransmitter analysis system.

The fifth method encompasses optical fiber biosensors, which operate as hybrid techniques incorporating other sensing approaches for detection or delivery. One example involves using optical fiber bioprobes in conjunction with the SERS technique, achieved by applying a functionalized layer of gold nanoparticles to the bioprobe’s tip and measuring the resulting SERS signal at the probe’s location. Similarly, a similar bioprobe can be employed in combination with fluorescence measurements, wherein the exciting light is injected into the sample, and the emitted fluorescence light is measured from the sample. Optical fiber bioprobes hold utility both in in vivo and in vitro analysis due to their versatile nature, much akin to electrochemical or microdialysis probes. Furthermore, the fabrication techniques for optical fibers are well-established, rooted in their extensive use in the realm of telecommunications, with similar principles applied to the creation of bioprobes. Overall, optical fiber bioprobes offer a promising avenue for in vitro miniaturized neurotransmitter sensing methods. Their compatibility with LoC or OoC devices could enable precise neurotransmitter measurements within samples, provided that the appropriate secondary sensing method is thoughtfully integrated into the device.

The sixth method falls under the classification of electromagnetic detection techniques, comprising LSPR and SERS measurement methods. Although these two techniques differ considerably, they share a common underlying principle. SERS is generally more prevalent than LSPR, offering a higher detection threshold and enhanced data resolution due to its distinct measurement approach. Nonetheless, SERS is a more intricate technique, demanding advanced analysis methods and instruments in comparison to the relatively simpler requirements of LSPR. LSPR is primarily utilized for in vitro analysis due to its inherent detection nature. For instance, a common LSPR detection technique relies on colorimetry measurements, which prove more challenging to implement in vivo compared to in vitro settings. Similarly, SERS also finds more frequent applications in vitro due to the precision demands of instruments for high-resolution data. Notably, recent research endeavors have extended SERS to in vivo neurotransmitter analysis by harnessing its strengths in combination with the capabilities of optical fiber bioprobes. Looking ahead, the simplicity and user-friendliness of LSPR render it more suitable for miniaturized in vitro neurotransmitter sensors, making it a feasible choice for integration within LoC or OoC devices. Although the potential application of SERS in a similar manner is not implausible, the adoption of LSPR appears more straightforward, offering high resolution and detection limits under appropriate conditions.

### 3.3. Discussion

The future of neurotransmitter sensing, and indeed many other biochemical analyses, lies in the evolution of in vitro sensing platforms that can overcome the limitations of in vivo animal models. This transition is partly driven by changing perspectives on animal testing in the public eye and within scientific communities. Stricter regulations are moving towards reducing the use of animals in scientific research. Additionally, flaws in animal models have been exposed when compared to human models, which they were meant to mimic.

Furthermore, in vivo analysis is inherently complex, presenting numerous challenges and uncertainties that are hard to control, leading to difficulties in reproducibility. To address these changes and challenges, a new era of systems that does not rely on animal models or in vivo techniques is required. In response, in vitro LoC and OoC devices have been identified as the preferred options moving forward. These platforms offer researchers the control and reproducibility that in vivo measurements often lack. They are also inherently simpler to use, potentially offering a more autonomous and user-friendly approach.

To create an effective in vitro LoC neurotransmitter sensing platform, an appropriate sensing technique should be chosen and integrated into the LoC platform. Not all neurotransmitter sensing techniques are suited for this application, as some require large and complex instruments and techniques. The ideal techniques for this purpose should be minimally invasive and simpler than others while maintaining a high degree of sensitivity and precision, as they were designed for in vivo applications. Such techniques would readily apply to in vitro LoC neurotransmitter platforms.

In terms of fabrication, cutting-edge techniques should be employed to create a LoC system that is compact, simple to use, and offers a high degree of sensitivity, precision, control, and reproducibility over experimental conditions.

The envisioned system that aligns with these requirements would be an in vitro-based LoC or OoC system. This experimental platform would consist of a miniaturized controlled receptacle where a neural matrix of neurons would be cultivated. Alongside it, a biochemical control platform equipped with various environmental sensors and a microfluidic system would monitor and nourish the neural matrix to replicate in vivo conditions, facilitating its growth and fulfilling its primary needs. On the other hand, a neurotransmitter sensing platform based on a specific sensing method, whether electrochemical or optical, would apply controlled and reproducible conditions to the matrix to monitor changes and collect data induced by these conditions.

The entire system would be compact and autonomous, with users only needing to set up the system for experiments and subsequently analyze the data to draw conclusions.

The creation of such a device is feasible using modern fabrication techniques. The electrical and mechanical aspects of the system are based on established techniques and are not challenging to apply. However, the real challenges lie in the sensing and incubation platforms. For the sensing system, work has already been done toward compact and autonomous neurotransmitter sensing systems based on techniques well-suited for in vitro models. Some examples of such systems can be found in Table 2. These systems can continue to improve in the future, increasing their sensitivity, selectivity, and miniaturization. In terms of the incubator, numerous technologies are available for creating such a system, many of which have been used for similar instruments presented in the literature. Given the wide variety of technologies, exploring existing options to identify their pros and cons will be essential for selecting the most suitable one.

Overall, there is confidence that the scientific community is moving toward a new era of in vitro biochemical analysis platforms. Researchers are actively working toward this goal. However, given the diverse range of sensing techniques, fabrication processes, and applications, there is no one-size-fits-all solution that will yield superior or faster results than others. Steady advances in various areas will collectively pave the way for true in vitro LoC neural sensing platforms, replacing current in vivo models and contributing to a deeper understanding of the brain. The future holds the answers to these unfolding developments.

## 4. Fabrication of LoC or OoC Type Device for the New Generation of In Vitro Sensors

For the next generation of in vitro sensors, a range of both new and existing technologies can be harnessed to design and fabricate integrated LoC or OoC devices. In this context, an optimal approach would involve incorporating the necessary sensing method along with a micro-incubator to cultivate and maintain viable cells throughout the experimental process. This comprehensive setup would enable in vitro experiments to encompass all essential elements required for studying cells directly within a controlled environment, facilitating autonomous monitoring and management of the entire experimental process.

This section offers a comprehensive overview of various fabrication techniques employed to integrate micro-incubators with sensing systems. These components, when combined, create a compact and autonomous in vitro LoC device that holds tremendous potential as the future of sensor technology. The subsequent exploration of these fabrication methods delves into the practical aspects of this innovation, illuminating the inherent possibilities stemming from the convergence of these advanced technologies.

### 4.1. $CO_{2}$ Based Incubators

An incubator serves as a controlled environment designed to provide the optimal conditions for the growth and thriving of cellular cultures. The growth of cells necessitates precise maintenance of factors such as temperature, humidity, and pH levels. This is achieved through a combination of elements within the incubator, including a heating mechanism, an air circulation system, and an array of sensors for continuous feedback.

To ensure proper pH control, the gas composition within the incubator plays a crucial role. Typically, the incubator atmosphere consists of air enriched with 5% CO_2_. The acidic nature of $CO_{2}$ interacts with the slightly basic growth medium, facilitating pH regulation through diffusion across a specialized membrane. By modulating the flow of gas, precise control over the pH of the medium can be achieved. Meanwhile, temperature regulation is achieved by modulating the heating elements through which the gas circulates, analogous to a conventional convection oven.

#### Control of pH Using a Physiological CO_2_/ ${\mathrm{HCO}}_{3}^{-}$ -Based Buffering Regime

Within culture chambers, a buffering system is employed to maintain pH stability. The CO_2_/ ${\mathrm{HCO}}_{3}^{-}$ buffering system is recognized as a pivotal extracellular buffer within bodily fluids [172]. In this system, CO_2_ functions as an acidic gas, while ${\mathrm{HCO}}_{3}^{-}$ acts as a soluble base.

The buffering capacity is achieved by introducing ${\mathrm{HCO}}_{3}^{-}$ salt into the medium. This salt reacts with the layer of CO_2_-enriched air above it, effectively regulating the pH within the incubator chamber. However, to ensure the stability of this system, accurate monitoring of pH levels is imperative. Spectrographic analysis serves as a valuable tool for this purpose. Specifically, a dye such as Phenol Red (PhR) is introduced into the growth medium, and its absorbance spectrum is subsequently analyzed using computational techniques. This analysis enables precise determination of pH levels based on the characteristic spectrum of the dye [172].

### 4.2. Materials for Incubator Fabrication

In the context of fabricating integrated micro-incubators, the selection of appropriate materials is of paramount importance. This section delves into the pivotal process of material choice, emphasizing their critical role in the successful construction of these advanced systems.

#### 4.2.1. PDMS as a Structural Material

Polydimethylsiloxane (PDMS), an organic polymer derived from silicon, holds a significant position in microfluidics applications. Its popularity stems from its suitability for prototyping, controllable mechanical properties, and biocompatibility. The cost-effectiveness of PDMS makes it an ideal candidate for rapid and economical micro-incubator development. Additionally, its affordability facilitates the use of disposable microfluidic systems, a feature particularly advantageous for single-use cellular culture applications, eliminating the complexities associated with cleaning intricate microfluidic structures. PDMS also permits the fabrication of three-dimensional components with more intricate internal structures than achievable with conventional materials like glass.

An illustrative instance of PDMS utilization is demonstrated in the incubator design by Blain et al. [173], where the incubator chamber is predominantly fashioned from PDMS. Notably, the only exceptions are the heating elements and the associated electronics responsible for temperature regulation. The latter, being non-contact components, and the former, constructed from silicon enveloped in aluminum for enhanced heating capabilities, are not in direct contact with the growth medium.

Furthermore, PDMS’s gas permeability allows for the creation of membrane elements, presenting opportunities for specialized incubator configurations. However, this permeability can pose challenges, particularly when dealing with intricate gas manipulation channels susceptible to external gas effects or leakage. Establishing a vacuum within a PDMS-constructed incubator chamber may prove infeasible. Nonetheless, strategic implementation of protective layers atop PDMS can enable vacuum conditions [174], enabling the realization of intricate PDMS-based designs involving complex gas control.

PDMS exhibits noteworthy temperature resistance, enduring temperatures up to 350 degrees Celsius when adequately cured [175]. However, its limited chemical compatibility hampers its application in settings involving organic solvents [175]. Similar constraints also apply to other polymers suitable for microfluidic devices.

#### 4.2.2. Glass as a Structural Material

Glass has a long-standing history as a foundational material for microfluidic channels. It was the primary choice before the rise of PDMS and remains relevant in certain contexts. Glass-based microfluidic channels are renowned for their transparency, an attribute that facilitates direct integration with microscopes or similar devices for real-time culture observation—a feature that holds particular appeal for micro-incubators.

Incorporating glass into incubator designs is not uncommon, often in conjunction with materials like aluminum, due to the complexities associated with fabricating intricate 3D glass structures [176]. The transparency of glass also allows for direct observation of cultures when coupled with a microscope. Although PDMS structures can also be used for this purpose to a certain extent, they tend to become contaminated by the culture media over time.

Glass microfluidic channels can be fashioned through lithography techniques similar to those employed in CMOS chip fabrication. The creation of 3D structures involves layering with thermal bonding [177]. However, these techniques are considerably intricate and demand advanced equipment. In contrast, PDMS molding offers a more practical avenue for prototyping applications.

A notable advantage of glass is its biocompatibility [178], rendering it suitable for prolonged cellular growth. The durability and moderate ease of cleaning associated with glass further bolster its appeal, making it a viable option for multi-use micro-incubator fabrication when structurally feasible.

It is important to note that glass-based incubators may still require membrane elements, typically based on materials like PDMS. By incorporating wear considerations of PDMS-based components into the design of glass incubators, a hybrid approach becomes viable. This hybrid strategy capitalizes on the strengths of both materials, facilitating glass cleanliness and PDMS replacement when necessary, therefore extending the device’s lifespan.

#### 4.2.3. Silicon Photonic Crystals-Based Micro-Incubators

An innovative approach to micro-incubator fabrication involves the utilization of electrochemical micromachining on silicon wafers, resulting in a technology that can be produced at a large scale and a remarkably low cost [179]. This method offers the potential to address various challenges associated with conventional micro-incubators, such as cost-effectiveness, bulkiness, and limitations akin to PDMS or glass-based alternatives. This advancement paves the way for numerous biomedical integrated solutions, particularly in the realm of in vitro LoC sensing systems.

The technique proposed in this context enables the creation of micro-incubators on a miniature scale. The process entails the formation of a periodic array of gaps within the silicon through electrochemical etching. These gaps serve as compartments for cellular cultures to thrive, fostering growth and development. The cultivated cells reside within these silicon gaps, which are filled with a suitable growth medium, and the pH can be regulated using the CO_2_-based method discussed previously.

By integrating an external temperature control unit, this technology has the potential to evolve into a microbioreactor, which can then be transformed into a fully functional LoC system applicable across a wide range of applications [179]. This innovative approach not only contributes to cost-effective and versatile micro-incubator technology but also underscores its potential integration into advanced in vitro sensing platforms.

#### 4.2.4. Thermoplastic Materials

Poly(methyl methacrylate) (PMMA) is a thermoplastic material frequently employed for microfluidic channel fabrication. Its properties make it a suitable choice for prototyping applications due to its ease of manufacture. Many techniques used to create microfluidic channels with PMMA can be adapted for other thermoplastics, such as cyclic olefin copolymer (COC) or polycarbonate (PC), which offer enhanced mechanical properties compared to PDMS, along with simpler manufacturing than glass.

One PMMA fabrication method involves maskless CO_2_ laser etching of the PMMA substrate to generate microchannels [180]. After etching, the piece is cleaned with acetone to remove debris, and channels are sealed by affixing a glass plate atop the PMMA using epoxy, resulting in a waterproof microfluidic cell.

Thermal nanoimprinting lithography (Thermal-NIL) is another method [181]. Here, a silicon wafer is etched using maskless UV lithography to create a stamp. The heated stamp is pressed onto a PMMA wafer to imprint features, and channels are sealed by thermal bonding between the PMMA substrate and another PMMA cover. This process is scalable if the stamp remains usable, enabling cost-effective replication of microfluidic cells.

A similar technique uses SU-8 on a glass substrate, patterned via standard lithography, to create a stamp for imprinting [182].

PMMA’s biocompatibility, resistance to temperature stress, minimal chemical reactivity, and limited interference with bioprocesses make it well-suited for micro-incubator fabrication, aligning it with the requirements of integrated in vitro sensing platforms.

#### 4.2.5. Choice of Material

The choice of material depends on the specific requirements of the application. Here are the key considerations for selecting the appropriate material for micro-incubator fabrication:

- PDMS is a versatile choice suitable for prototyping, disposable applications, and scenarios where cost and simplicity are important. It allows for rapid fabrication and is well-suited for one-off or short-term experiments. PDMS is ideal when the focus is on quick testing and proof-of-concept.
- Glass is a premium option, offering excellent biocompatibility, optical transparency, and durability. It is a suitable choice for applications where reliability, long-term culturing, and high-quality observations are essential. Although it is more challenging to manufacture complex structures, glass-based incubators provide a cleaner environment for extended cell culture.
- Thermoplastics, such as PMMA, COC, and PC, strike a balance between PDMS and glass. They are suitable for applications where moderate production scalability, cost-effectiveness, and reasonable longevity are desired. Thermoplastics offer better mechanical properties than PDMS and can provide a compromise between ease of fabrication and quality of incubator structure.

In summary, PDMS is advantageous for rapid prototyping and disposable setups, glass excels in long-term and high-quality culturing environments, and thermoplastics provide a middle ground between ease of fabrication and durability. The choice should align with the goals of the experiment, the level of precision required, and the resources available for fabrication.

### 4.3. Designs of Micro-Incubators: Portable Circular Incubators

One prevalent design for microscope-mounted micro-incubators is a circular geometry, chosen to align with the circular lens of the microscope for convenient observation. The circular architecture not only simplifies the manufacturing process but also enhances temperature and pressure control due to the even distribution of cellular culture within the circular chamber [176].

To ensure controlled cell culture, maintaining proper pH levels is essential. The micro-incubator described by Ince, C. et al. [176] employs the same $CO_{2}$ -based system described in Section 4.1 for pH control.

Recent advancements in fabrication techniques have enabled the creation of circular micro-incubators at a significantly lower cost while achieving even higher levels of performance and control [183]. Temperature fluctuations can be minimized to as low as 0.05 degrees Celsius using a Peltier module thermally connected to the incubator’s base, along with a digital thermometer for precise temperature monitoring.

The incorporation of a Peltier module allows for temperature regulation both above and below ambient temperature, providing greater flexibility. The module is designed to handle transient temperature changes and can compensate for them in less than 5 min.

Such micro-incubator designs effectively accommodate both 2D monolayers and 3D tissue samples, making them versatile tools for various cell-culture applications.

### 4.4. Designs of Micro-Incubators: On-Chip Incubators

#### 4.4.1. On-Chip Carbon Dioxide Generator

To eliminate the need for an external CO_2_ supply, which typically involves a large compressed tank and a dedicated inlet controller, an onboard solution can be developed. Instead of relying on an external CO_2_ source, an on-chip micro-heater can be employed to heat sodium carbonate ( ${\mathrm{NaHCO}}_{3}$ ) and generate CO_2_ gas for pH regulation [184]. The micro-heater is positioned beneath the culture media, allowing the generated gas to rise through a PDMS membrane to control the culture’s pH.

It is worth noting that this method of CO_2_ generation requires the micro-heater to be fueled with sodium carbonate. Additionally, micro-pumps are needed to draw and control the gas flow to accurately regulate the pH.

Enhancements to this micro-heater CO_2_ gas source have shown improvements in the stability and viability of cell cultures over time [185]. One significant advancement involved using a mixture of sodium bicarbonate and sodium carbonate, resulting in more stable pH regulation. This upgraded system enabled the design of a pocket-size micro-incubator that operates without the need for pH feedback control. With no active pH regulation, the incubator can run on batteries for several days without external intervention.

In a notable application, epithelial cell cultures were maintained for 17 days using this system [185]. This technology opens possibilities for mailing cell cultures, allowing remote locations to send samples to main laboratories while preserving cell viability. The design focuses on portability, with heating element power consumption being a primary limitation. At around 500 mW power consumption, the incubator can realistically maintain cell cultures for up to a week during regular shipping. Further optimization of power consumption could potentially extend the stable culture period.

#### 4.4.2. Real-Time Sampling and Observation

Another challenge in micro-incubators and cell culture is the real-time sampling and visual monitoring of cultures. A solution involves integrating an array of electrodes and optical sensors into the incubation chamber for continuous monitoring of cell growth [186]. The design includes micromachined microprobes, 10 platinum electrodes, 2 pH electrodes, and a temperature sensor, all of which allow active feedback. A video camera positioned above the incubator connects to a computer for optical monitoring, while a microfluidics system enables media insertion into the cell culture [186].

This integration enables the control of key parameters essential for observing cellular growth, making it suitable for maintaining and testing complex organisms that require real-time monitoring. The fabrication of such a device can be accomplished using silicon-based substrates. Etching the silicon substrate can create space for electrodes, the incubation chamber, and pH probes, providing a versatile platform for various applications.

Potential applications of this device include medical, pharmaceutical, and toxicity monitoring in samples, as well as real-time monitoring of other biochemical processes based on electrochemical detection, such as neurotransmission.

### 4.5. Fabrication Processes

#### 4.5.1. PDMS-Based Micro-Incubators

Due to its biocompatibility and utility in microfabrication techniques, PDMS is frequently employed in soft lithography to create micro-incubators. Notably, studies have demonstrated the successful fabrication of entire micro-incubators using PDMS as the primary material [173].

All PDMS structures are crafted using soft lithography, a technique that facilitates the curing of thermocurable polymers through additive manufacturing. This process involves creating a mold by adding layers of material where needed. To generate cavities such as those required for microfluidics channels, an additional material is introduced to create spaces for the deposition of the mold material. Subsequently, the original material is dissolved using a solution that does not affect the primary mold, leaving behind the desired structures. The mold is then filled with the polymer, after which the mold itself is dissolved in a solution, resulting in the final product, a precisely shaped PDMS component [187]. The molding process is described in detail by Rodrigue, H., Bhandari, B., Wang, W., and Ahn, S.H. [187]. This approach enables the fabrication of intricate and complex 3D PDMS structures, a material known for its biocompatibility [188].

For PDMS structures of less intricate complexity, more expedient molding processes are available [189]. This method involves a hybrid structure, commencing with a silicon pad onto which photoresist is applied through spin coating, much akin to the process employed with doped silicon wafers. The channel structure is created by subsequently removing the photoresist through plasma treatment, leaving a negative imprint. PDMS is poured and cured onto this negative imprint, and once fully cured, the PDMS structure is peeled off and attached to a PDMS plate featuring access ports for channels. This fabrication process facilitates more rapid automated production as PDMS only requires curing once. Additionally, the peel-off method eliminates the need to dissolve the inner canal, rendering it a cost-effective alternative.

#### 4.5.2. Tunable Permeability PDMS Membranes

PDMS membranes provide precise control over gas exchange within PDMS structures. This characteristic can be effectively harnessed to regulate CO_2_ concentrations within culture chambers of micro-incubators. This innovative approach offers enhanced control over cellular growth by enabling the regulation of gaseous environments without direct gas injection, which could otherwise lead to media displacement and turbulence in the culture medium—particularly problematic in specific incubator designs.

Although a method for countering the gas permeability of PDMS by sealing it with a coating was previously discussed [174], there are instances where increased or tuned gas exchange is required. For such applications, a PDMS membrane with varying gas permeability was designed [190]. This is achieved by altering the ratio of polymer to curing agent in the PDMS composition. This introduces a novel parameter for consideration during incubator design. However, such alterations require careful design considerations, as changes to PDMS composition can impact mechanical properties, potentially hindering the fabrication of certain structures.

This alteration in permeability is largely attributed to silicon chains. The inherent flexibility of silicon-oxygen (Si-O) chains creates spaces that enable gas molecules to permeate the polymer matrix. The movement of these Si-O chains displaces trapped gas molecules, allowing them to traverse the membrane [190].

To create these tunable membranes, varying ratios of polymer to curing agent (ranging from 5:1 to 20:1) can be employed. These membranes are crafted using the spin coating method. Membrane thickness is determined through the spin coating process, while the area is defined through cutting and peeling post-thermal hardening. The PDMS membrane is assembled between a hard PDMS interconnect and a glass substrate, resulting in a dead-ended microfluidic channel tailored for permeability testing. Gas is pushed through the channel at a controlled rate using a pump. By measuring the resulting pressure and volume of escaping gas, the membrane’s permeability can be determined using Equation (1) [190].

(1) $$P=\frac{\nu \delta }{At(p_{1}-p_{0})}$$

where *P* represents permeability, ν denotes gas volume through the membrane, δ signifies membrane thickness, *A* represents area, *t* stands for time, $p_{1}$ indicates high partial pressure, and $p_{0}$ signifies low partial pressure.

#### 4.5.3. Rapid Prototyping of 3D Systems

A method for fabricating complex 3D PDMS components involves a combination of conventional spin coating and lithography techniques in a layered fashion, termed the “membrane sandwich” [191]. This technique entails the creation of two membranes through traditional means, followed by sandwiching them between thicker slabs for structural reinforcement. These robust slabs, often made of glass, are drilled at appropriate positions to allow fluid passage between layers within the 3D structure, therefore introducing a new dimension of complexity. This novel approach can be likened to the distinction between single-layer and double-layer printed circuit boards, therefore expanding the realm of possibilities.

Modifying the mechanical properties of the PDMS structure makes it feasible to stack PDMS membranes without the need for additional structural support layers. This advancement paves the way for entirely PDMS-fabricated incubators, excluding the electronic peripheral components.

#### 4.5.4. Comparison of Fabrication Processes

Just as there exists diversity among types of incubators, the fabrication processes for these incubators are equally varied. The selection of a particular fabrication method may be influenced by the specific type of incubator under consideration, and in some instances, a certain method might even be essential. In the pursuit of engineering a micro-incubator for research, the optimal fabrication process aligns with the desired performance metrics and versatility demanded by the application. Table 3 presents a concise overview of the technologies discussed in the preceding sections.

### 4.6. Packaging and Integration of the LoC System

To realize a fully functional LoC system, the integration of a functional micro-incubation component with its associated peripherals is imperative. This integration facilitates the consolidation of various sub-systems into a singular instrument, enhancing portability and reducing the overall footprint compared to modular arrangements. A plethora of methodologies exist for achieving this integration.

One notable approach involves the fabrication of the micro-incubator directly into the sensing system. For instance, a complementary metal-oxide-semiconductor (CMOS) die can be repurposed as an electrode array for the detection of subtle electrical signals. In this setup, the micro-incubator chamber or channel can be fashioned from materials such as PDMS, glass, or other polymers seamlessly onto the surface of the CMOS chip. This design philosophy revolves around the amalgamation of integrated circuitry with microfluidic components to enable advanced sensing applications. By situating on-chip amplifiers beneath the sensing electrodes, the distance traveled by the signal from its source is minimized, leading to heightened signal-to-noise ratios (SNRs). It is crucial to note, however, that this fabrication approach entails certain trade-offs. Notably, the direct contact of the electrodes with the liquid medium within the incubator raises challenges in packaging to prevent issues such as leakage.

The integration of diverse technologies can manifest in numerous forms, contingent upon the specific experiment and application demands. Datta-Chaudhuri, T., Abshire, P. and Smela, E. [192] describe the predominant packaging techniques employed in microfluidic LoC systems in their work. Although the configurations of these packages can differ based on the unique application needs, the underlying fabrication process tends to maintain a degree of similarity across various packaging designs. This streamlined process stands as a formidable contender at the forefront of current practices due to its inherent simplicity, rendering it an elegant solution to the intricate packaging challenges associated with LoC systems.

### 4.7. Technology Comparison

In the realm of designing LoC systems with integrated micro-incubators, several critical factors come into play, including size, ease of use, and fabrication complexity. The dimensions of the incubator hold significant importance, influencing factors such as culture observation capabilities, mobility, and fabrication intricacy. Larger incubators generally afford enhanced culture observation, often featuring modular designs that are user-friendly and straightforward to fabricate, albeit at the expense of portability. Conversely, smaller incubators offer cost-effective manufacturing processes and precise operations, albeit with potential drawbacks in terms of culture size and versatility.

For a comprehensive overview of different technologies pertinent to the LoC micro-incubator systems, refer to Table 4, which presents summarized information delineating their unique characteristics and attributes.

## 5. Conclusions and Future Perspectives

In this comprehensive review, we have meticulously examined the recent strides in the field of neurotransmitter sensing, with a particular emphasis on their applications in both in vitro and in vivo contexts and their potential integration within LoC devices. We evaluated many different neurotransmitter sensing technologies, shedding light on their capabilities, limitations, and prospects.

### 5.1. Recent Advances and Trends

Our exploration of neurotransmitter sensing explored various domains. In the realm of medical imaging, significant progress has been observed, particularly in utilizing established techniques for monitoring neurotransmitters within neurodegenerative disease studies. Recent investigations have also delved into the impact of the SARS-CoV-2 virus on the brain, shedding new light on this critical area. Moreover, innovative amalgamations of imaging methods such as magnetic resonance spectroscopy (MRS) and positron emission tomography (PET) have been explored to glean comprehensive neurotransmitter maps. Although the miniaturization of these imaging techniques for LoC integration is yet to be realized, they remain pivotal in their conventional applications.

Electrochemical sensing, propelled by advanced materials like carbon-fiber-based bioprobes, has garnered substantial attention. These novel probes offer the dual advantage of compact size and heightened data resolution, rendering them invaluable for voltammetry and amperometry analyses of neurotransmitters. Encouragingly, the synthesis of these advanced probes presents an enticing synergy with LoC platforms, capitalizing on their portability and ease of use.

The dialysis approach to neurotransmitter analysis, facilitated by microdialysis probes, has sustained steady progress. Notably, the introduction of solid-state microprobes holds promise for the future, offering smaller form factors and enhanced usability. However, the pivotal challenge in this domain lies in the need for secondary analysis techniques such as high-performance liquid chromatography (HPLC) or mass spectrometry (MS), which have exhibited unmatched sensitivity and selectivity but have yet to be miniaturized for LoC integration.

Advancements in optical techniques have emerged as a formidable alternative, with fluorescence, chemiluminescence, surface-enhanced Raman spectroscopy (SERS), and localized surface plasmon resonance (LSPR) demonstrating remarkable sensitivities and selectivities. These techniques, whether harnessed alongside bioprobes or optical imaging, offer promising avenues for neurotransmitter detection. The pinnacle of these developments resides in the creation of optical fiber biosensors, capable of integrating diverse techniques to facilitate accurate neurotransmitter detection at targeted sites.

### 5.2. Future Perspectives

The future of neurotransmitter sensing seems very different than what is present at the moment. Amid shifting attitudes toward animal testing, in vitro analyses are poised to ascend, potentially relegating in vivo methodologies to a last resort. The evolution of in vitro alternatives assumes paramount importance to sustain neurotransmitter analysis and broader physiological studies, requiring platforms such as LoCs or OoCs devices to faithfully emulate in vivo conditions. The relentless pursuit of this goal is demonstrated in the progress of brain organoid models, offering promising 2D and 3D neuronal matrices for neurotransmission and brain chemistry analysis.

The trajectory of progress converges on the imperative development of compact, low-cost, and autonomous platforms. Herein lies the potential of LoCs as the future of neurotransmitter sensing. These platforms possess the dual advantage of obviating the need for animal models in in vivo analysis and conferring autonomy and controllability, facilitating the replication of experiment characteristics across trials. The advent of existing technologies that enable the amalgamation of artificial organs with direct sensing and monitoring techniques on a single platform underscores the capability of LoCs. As the methods of neurotransmitter analysis shift toward in vitro paradigms, a future where a micro-incubator chamber fosters the growth of disease-specific neurons alongside an integrated fiber optic bioprobe equipped with LSPR or SERS technology, all seamlessly controlled and analyzed by an onboard computer, seems feasible.

Although these goals promise a transformative future, there are still many difficulties to overcome, which would require the expertise of multidisciplinary collaboration to overcome. The pathway to realizing these platforms is obtainable, promising to revolutionize not only biochemical analysis but also democratize access to sophisticated research instruments for remote laboratories or research institutes that might not have access to advanced machines for those complex manipulations.

## Figures and Tables

**Table 1:** Neurotransmitter sensing methods: a comparison for a new area of miniaturized sensors.

Technique	Ease of Use	Location	Potential for Miniaturization in the Short Term	Reference
MRI, MRS, PET, SPECT	Low	in vivo ≥ in vitro	Low ^†^	[12,13,14,15,16,17,18,19,20,21,22]
CV, FSCV, Amperometry	High	in vitro + in vivo	High	[3,23,24,25,26,27,28,29,30,31,32,33,34,35,36,37,38]
Microdialysis	High *	in vivo ≥ in vitro	High *	[11,39,40,41,42,43,44,46,47]
Fluorescence	Medium	in vitro	Medium	[48,49,50,51,52,53,54,55,56,57]
Chemiluminescence	Medium	in vitro	Medium	[58,59,60,61,62,63,64]
Optical Fiber biosensors	Medium	in vitro + in vivo	High	[66,67,68,69,70]
LSPR	High	in vitro	Medium	[68,106,107,108,109,110,111,112,113]
SERS	Low	in vivo ≤ in vitro	Medium	[30,69,71,72,73,74,75,76,77,78,79,80,81,82,83,84,85,86,87,88,89,90,91,92,93,94,95,96,97,98,99,100,101,102,103,104,105]

* With the usage of a secondary analysis method. ^†^ Miniaturization of PET and SPECT instrument were demonstrated previously but are not common [114,115,116].

**Table 2:** Report of different microfabricated LoC or OoC types of devices for biochemical studies. The application of each device, the sensing technique employed, and the measurement resolution are presented.

Authors	Application	Sensing Method	Detection	Reference
Ndyabawe et al.	Brain-on-a-chip	Fluorescence	Imaging	[146]
Uzel et al.	Controlled concentration gradient in cell matrix	Fluorescence	Imaging	[147]
Park et al.	AD pathogenesis	Fluorescence	Imaging	[148,154]
Yong et al.	Axon degeneration	Fluorescence	Imaging	[149]
Bologini et al.	PD-specific neurons	Fluorescence	Imaging	[150]
Liu et al.	Epilepsy-on-a-chip	Voltammetry	Electrical recordings	[151]
Shin et al.	Blood-Brain Barrier	Fluorescence	Imaging	[152]
Majumdar et al.	Neural co-culture	Fluorescence	Imaging	[155]
Li et al.	Synaptic release	Amperometry	1 μ M NE	[156]
Kundu et al.	3D Printed, 3D Microelectrode Arrays	Voltammetry	Electrical recordings	[157]
Hyung et al.	Peripheral nervous system–on–a–chip	Fluorescence	Imaging	[158]
Yu et al.	Dopamine uptake	Voltammetry	30 nM DA	[159]
Senel et al.	Dopamine detection	Amperometry	0.1 nM DA	[160]
Taylor et al.	Neuronal culture device	Fluorescence	Imaging	[161]
Croushore et al.	neuropeptide signaling	External MS	S/N ≥ 5	[162]
Sharma et al.	3D functional human peripheral nerve	Voltammetry	Electrical recordings	[163]
Johnson et al.	3D printed neurons-on-a-chip	Fluorescence	Imaging	[170]

**Table 3:** Summary of different manufacturing technology employed for micro-incubator fabrication.

Technique	Application	Advantages	Disadvantages	Reference
Hybrid CMOS/PDMS Microsystem	A low-cost centimeter-scale disposable microdevice for cell culture and incubation	Less costly than CMOS alternative, biocompatible, ease of use	Single-use-only, fabrication of the mold and every unit is difficult	[173]
3D soft lithography	Fabrication of complex 3D PDMS parts	Allows fabrication of complex PDMS structures, biocompatible	Complex fabrication process, Single-use-only, not scalable	[187]
Positive CMOS mold fabrication for PDMS molding	Fabrication of a microfluidic platform to study single cells and multicellular aggregates in 3D	Well-established micromachining techniques, simple molding process, biocompatible	System needs to be sealed using glass plates, single-use, moderately scalable	[174,188,189]

**Table 4:** Incubator technology and size comparison.

Technology	Dimensions	Application	Advantages	Disadvantages	Reference
Conventional micro-CO_2_-incubator for use on a microscope	Compact	Culture observation, Numeric vision analysis	Glass top for visibility, good temperature and pressure control	High cost, harder pH control	[176]
New compact micro-CO_2_-incubator for use with an imaging device	Compact	Multi-culture control and monitoring	Glass top and bottom for visibility, good temperature, and pressure control, low-cost fabrication, multisample	High complexity	[193]
Microfabrication of a micro-incubator for cell and tissue imaging	Compact	Observe cell monolayers and tissue samples in a changing controlled environment	±0.05 °C temperature control, heating or cooling, 2D or 3D samples	Medium composition change, membrane change every 10 experiments	[183]
Micro Cell Incubator with On-Chip Integrated CO_2_ Generator as a Self pH Controller	On-Chip	Cellular culture in remote areas, low maintenance applications, preservation of cellular cultures for transport	Internal CO_2_ production, up to 9 cell chambers	Lesser pH control than incubators with CO_2_ tanks	[184]
On-chip CO_2_ incubation for pocket-sized microfluidic cell culture	On-Chip	Allows shipment of cellular cultures from remote areas	On-chip CO_2_ production, very low maintenance, maintain cultures unattended for up to 17 days	No pH feedback control, power consumption issues for portability	[185]
Miniaturized Integrated Platform for Electrical and Optical Monitoring of Cell Cultures	On-Chip	Real-time sampling, Pharmaceutical research, and drug monitoring for personalized treatment	Allows precise observation of cellular growth, modular platform for computer analysis	Requires proper control and understanding of the platform	[186]

## Data Availability

Data are contained within the article.
